# Mapping Urban Environmental Noise Using Smartphones

**DOI:** 10.3390/s16101692

**Published:** 2016-10-13

**Authors:** Jinbo Zuo, Hao Xia, Shuo Liu, Yanyou Qiao

**Affiliations:** The Institute of Remote Sensing and Digital Earth, No. 20 Datun Road, Chaoyang District, Beijing 100101, China; zuojb@radi.ac.cn (J.Z.); liushuo@radi.ac.cn (S.L.); y56yqiao@irsa.ac.cn (Y.Q.)

**Keywords:** noise map, smartphone, participatory sensing, spatial interpolation

## Abstract

Noise mapping is an effective method of visualizing and accessing noise pollution. In this paper, a noise-mapping method based on smartphones to effectively and easily measure environmental noise is proposed. By using this method, a noise map of an entire area can be created using limited measurement data. To achieve the measurement with certain precision, a set of methods was designed to calibrate the smartphones. Measuring noise with mobile phones is different from the traditional static observations. The users may be moving at any time. Therefore, a method of attaching an additional microphone with a windscreen is proposed to reduce the wind effect. However, covering an entire area is impossible. Therefore, an interpolation method is needed to achieve full coverage of the area. To reduce the influence of spatial heterogeneity and improve the precision of noise mapping, a region-based noise-mapping method is proposed in this paper, which is based on the distribution of noise in different region types tagged by volunteers, to interpolate and combine them to create a noise map. To validate the effect of the method, a comparison of the interpolation results was made to analyse our method and the ordinary Kriging method. The result shows that our method is more accurate in reflecting the local distribution of noise and has better interpolation precision. We believe that the proposed noise-mapping method is a feasible and low-cost noise-mapping solution.

## 1. Introduction

With the development of society and economy, noise pollution has become a new challenge for sustainable development in urban areas. In [1], the European Environment Agency stated that “noise pollution is a major environmental health problem in Europe. Environmental noise causes at least 1000 cases of premature death in Europe each year; over 900,000 cases of hypertension are caused by environmental noise each year”. Estimates have shown that approximately 20 million adults complain about environmental noise, more than 8 million people suffer sleep disturbances from environmental noise, and 43,000 cases of hospital admissions are caused by noise pollution each year in Europe [1]. Approximately 15% of the U.S. population aged 20–69 (26 million people) may have suffered from noise-induced permanent threshold shift caused by excessive exposure to workplace or leisure noise [2]. This problem is also pressing in developing countries [3].

Noise monitoring was usually carried out in certain regions in the past, and it is usually difficult to completely and accurately reflect the urban noise pollution in a large area. Noise mapping is a useful and effective assessment method, which can be combined with acoustics, geographical information system (GIS), and computational modelling to visualise the different regional exposure to noise pollution, statistics of affected population, and different noise source contribution analysis. Noise map is a useful tool in creating a government noise-control plan. It also provides an available method for the public to identify the environmental noise level around them.

Owing to the advantages of noise maps, they have attracted widespread attention nowadays and have been widely applied in many countries and regions [4,5,6,7]. In [8], the European Noise Directive requires all member states to prepare and publish noise maps and noise management action plans for large cities every five years, and the noise maps should be incorporated in the European Environment Agency’s ReportNet system. In [9], the authors inferred a fine-grained noise-mapping situation (consisting of a noise pollution indicator and a composition of noises) at different times of a day for each region in New York City using 311 complaint data together with social media, road network data, and points of interests. Some other available noise-mapping methods and techniques based on social media data have been proposed, such as the integration of soundscape with noise mapping in [10] and the study on the relationships among picture tags, emotions, and soundscapes in [11].

For these types of noise maps, three major data sources are available: (1) simulations based on inputs such as traffic-flow data, road or rail type; and vehicle type and (2) noise monitoring stations; (3) social media data. For the first, a simulation approach is extremely useful because it allows assessment of background noise in the absence of physical data with adequate granularity [12]. However, this method suffers from some problems. The most important of these problems is that the types of data sources are limited. This approach does not consider incidental sounds such as those generated by traffic jams, construction works, assembled crowd, and other unexpected sounds. As widely known, these sounds are also a disturbance to local dwellers. On the other hand, the collection of such input data is very expensive, which causes longer update intervals of these maps [13]. In addition, because the propagation of sound is greatly influenced by buildings and public facilities, these simulation approaches have difficulty in depicting the noise level of corners in urban areas.

For the second, although noise monitoring stations can achieve a high level of precision, the noise nevertheless dramatically varies with the spatial variation. Deploying millions of these stations to measure finer grained environmental noise in every nook and cranny of cities is impossible [9]. Maybe low-cost wireless sensing units have been already proven feasible for noise and other environmental data, such as in [14,15], but the problem is the feasibility in technology does not mean that we can acquire the data from these sensors easily. For example: in Beijing, with more than 20 million of population, and developed advanced transport system, it is not very convenient to learn sound level of environmental noise from public channel. We are also not sure whether this kind of sensing network is established. For the third, it is not a method to monitor noise relying directly on measurements and not the original intention of our study.

The lack of convenient and low-cost noise-mapping solutions with certain precision is always a problem. Nowadays, smartphones experience high adoption rates. Smartphones are personal devices carried all day long, which are equipped with different sensors and connected to the Internet. They are a perfect platform for sensing the environment [16]. Some researchers are searching for a new solution to create noise maps using smartphones in an attempt to reduce the cost of noise mapping and to improve their refresh frequency.

NoiseTube generates one map per measurement track using coloured dots for each individual measurement [12,17]. NoiseTube focuses on the culture of democracy and citizen science, where people can measure the noise they are exposed to in their daily environment. In this manner, they can create collective noise maps by sharing citizen measurements and personal annotations. Ear-Phone investigates the use of different interpolation and regularisation methods to address the fundamental problem of recovering a noise map from incomplete and random samples obtained by crowdsourcing data collection [13,18]. Ear-Phone leverages context-aware sensing to detect the status and placement of the phone to detect whether the phone is used for communication, whether the carrier is talking with others or passing through a conversation area, or whether the phone is held in the hand. NoiseMap is an application that gathers data on loudness and transfers them to da_sense. da_sense is an open urban sensing platform that allows users to access and control their data and to generate real-time noise maps and data graphs [19]. In [20], a spatial data infrastructure based on the open-source tools named OnoM@p (Institute for Research on Urban Sciences and Techniques, Nantes, France) is presented, which relies on participatory smartphone-based noise measurements. It aims to offer a framework for capitalizing on crowd noise data recorded by inexperienced individuals. In [21], the study offers a method of estimating noise levels and black carbon by the combination of mobile and fixed-station measurements. However, although these studies provide available methods to map noise, three major limitations remain.

1. The calibration of smartphones used to measure noise requires some operations, which may lead to a high volume of workload when the number of phones increases.
2. In these studies, the volunteers should always carry the phones in their hands because putting the phones in their trouser pockets, bags, or belt pouches may result in large errors, which would be ineffective in long-term data collection.
3. Because infinitely increasing the spatial density of measurements is impossible, a suitable interpolation method is needed to map noise. Owing to the spatial heterogeneity of noise distribution, the spatial interpolation method may be influenced by this heterogeneity. However, none of these works tried to solve this influence.

To address the abovementioned problems, the current paper proposed a low-cost and easy noise-mapping method using smartphones with additional microphones. The goals of the present study are as follows: a convenient calibration method using smartphones to measure environmental noise, feasibility of using additional microphone attached to the phone for noise measurement, and a suitable spatial interpolation method.

In this paper we proposed the following solutions: firstly, in order to achieve certain precision of the noise measurement by smartphones, we designed a set of methods to calibrate the smartphones and the calibration process can be done by the software. Secondly, we confirmed the necessity of attaching an additional microphone with a windscreen to reduce the effect of wind; as a result, we can release our hands and can put the mobile phones in our pocket which will bring great conveniences to the users. Thirdly, we proposed the region-based noise-mapping method, which is based on the distribution of noise in different region types tagged by volunteers, to interpolate and combine them to reduce the influence of spatial heterogeneity and improve the precision of noise mapping. We analyzed the interpolation results of our method and the ordinary Kriging interpolation method. The result shows that our method is more accurate in reflecting the local distribution of noise and consequently has a better interpolation precision.

The remainder of this paper is organised as follows: in Section 2, we detail the theory and techniques used in the proposed method. In Section 3, we outline the experimental process using the proposed method. We describe and analyse the results in Section 4. In Section 5, we present our concluding remarks.

## 2. Methods

This section describes the methods of mapping environmental noise using mobile phones.

### 2.1. Skeleton

**Figure 1:**
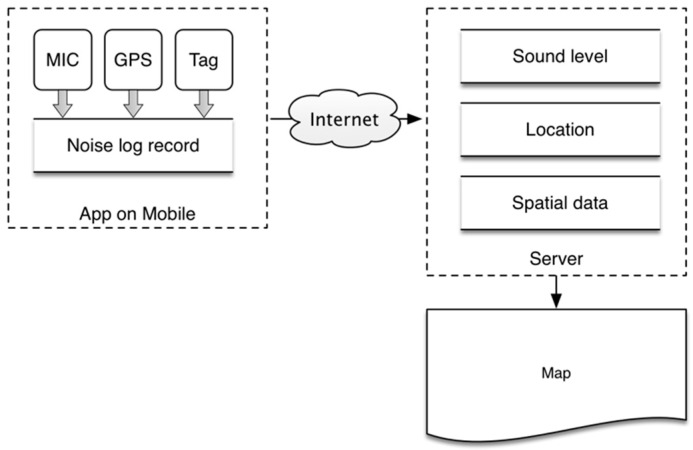
Skeleton of the noise-mapping prototype system.

After the calibration of the smartphones needed in the task, mapping the environmental noise can be done using our noise-mapping prototype system whose skeleton is shown in Figure 1. Using mobile phones, our application collects noise data through a microphone and obtains the location of the collection using Global Positioning System. Then, the raw data are processed and incorporated into noise log records with a timestamp. The records are transmitted through wireless Internet to our server. The server draws the noise maps using the noise and location data from the phones, together with the locally stored spatial data.

### 2.2. Measuring Noise

To measure noise using mobile phones, the equipment needs to meet the following requirements [12,13]: (1) A-weighting; (2) computation of *L_Aeq_* over arbitrary time intervals; (3) calibration; (4) procedure on how to carry the phones; and (5) wind protection. Requirements (1) and (2) correspond to the international standard for sound level meters (SLMs) [22]. We present our proposed rapid calibration method to satisfy requirement (3) in the next sections. An additional microphone is used to satisfy requirements (4) and (5).

#### 2.2.1. Additional Microphone

Although additional microphones will slightly increase the cost of smartphones, we use them for three reasons:

(1) Benefit for long-term data collection

Research conducted by Nokia [23] suggests that people tend to carry their mobile phones in their trouser pockets, bags, belt pouches, or hands. Expecting that volunteers will always carry their phones in their hands with the microphones correctly positioned to sample ambient noise would be unrealistic [13]. In the current study, we use additional microphones with extension windscreens, cords, and clips. The microphone can be pinned to the clothing of the volunteers. In this manner, the volunteers would be able to carry the mobile phones in their trouser pockets, bags, belt pouches, or hands without affecting the measurements. The measurement will not also be affected by the movements of the volunteers.

(2) Reducing the effect of environmental wind

Noise measurement should be taken on a day with calm to gentle breeze and without rain. Some conditions that must be avoided are strong wind (generally, we do not perform measurements if the wind is stronger than 5 m/s) and during rain [24]. Windscreens need to be installed on the devices when it is windy. The additional microphone can have a windscreen, but the built-in microphone cannot. An additional microphone with a windscreen can greatly reduce the wind effect.

(3) Facilitating measurements while moving

In traditional noise measurements, the SLM is fixed on a bracket. However, for environmental noise measurements using smartphones in participatory sensing [12], the volunteers cannot stay at the same place for a long time and will not be carrying a bracket with them to install the SLMs and other equipment. Some volunteers would be interested in collecting data when walking or even riding a bicycle. The wind caused by these activities will affect the measurements of environmental sound level. We believe that an additional microphone with a windscreen can minimise these effects.

#### 2.2.2. A-Weighted Equivalent Continuous Sound Pressure Level

Noise level is measured in terms of the A-weighted equivalent continuous sound level or *L_AeqT_*. A-weighting is commonly used frequency weighting for environment noise measurement because it can reflect the loudness perceived by a human being [22,25].

The sound pressure level is captured by a microphone as an induced voltage. To measure noise using a smartphone, firstly, we need to obtain induced voltage *v*(*t*) from the microphone sensors during time *T*. Then, *v*(*t*) should be processed using an A-weighted digital filter. After filtering, we can acquire the induced voltage using A-weighted *v_A_*(*t*). The A-weighting filter is standardised in the International Electrotechnical Commission (IEC) document [22]. The A-weighted equivalent sound level is expressed as follows:

(1) $$L_{AeqT}=20lg\left\{{\left[\left(1/T\right)\int_{0}^{T}v_{A}^{2}\left(t\right)dt\right]}^{1/2}/v_{0}\right\}$$

where *v*_0_ is the reference induced voltage. The standard reference pressure is 20 μPa [22]. However, for different smartphones, we do not know what input voltage corresponds to this reference pressure, i.e., what *v*_0_ value corresponds to 20 μPa. Because *v*_0_ is a fixed value, we can express *L_AeqT_* as follows:

(2) $$L_{AeqT}=10lg\left[\left(1/T\right)\int_{0}^{T}v_{A}^{2}\left(t\right)dt\right]+C.$$

Calibration constant *C* can be determined from experiments, as discussed in the next section.

#### 2.2.3. Calibration

According to Section 2.2.2, we must empirically determine the value of *C* in Equation (2). To simplify the problem, a linear model between the induced voltage from the phones and the measurement from the SLM is assumed. We let:

(3) $$V_{p}=10lg\left[\left(1/T\right)\int_{0}^{T}v_{A}^{2}\left(t\right)dt\right]$$

Then, we can establish the relationship between *V_p_* (the measurement from the phone) and *V_s_* (the measurement from the SLM) as:

(4) $$V_{s}=V_{p}+C+\varepsilon$$

where *ε* is the error. Thus, the value of *C* can be calculated.

A common method for obtaining *C* is by recording the phone responses to different sound levels. Along with the measurements by an SLM or other devices, linear interpolation is used to determine *C* [12]. However, the measurement process under different sound levels must reduce the influence of the background noise. With the increase in the number of phones, this process would incur a high volume of workload. For participatory sensing, convenience is more important if the required precision can be satisfied. Thus, we design a more convenient method to calibrate the mobile phones for noise measurement in which the calibration process can be programmed.

Similar to a related work [13], we use the freely available Audacity [26] software to construct a calibration tone. This tone is made up of two 4-min 1-kHz sinusoidal tones and silence that spans for 2 min (We use a pure tone for calibration, since standard calibration process uses one single frequency, namely 1 kHz). The amplitude scale of these sinusoidal tones is set from 0 to 0.6 for two reasons. On the one hand, we can achieve the offsets under different sound pressure levels and obtain the full response of the phones at different sound levels. On the other hand, when the response data are converted to the time series-sound level format, the data will be displayed in special shapes, including arched and inflection points. All of these obvious features will greatly help us and our programs make comparison of the SLM and the mobile phone shapes.

**Figure 2:**
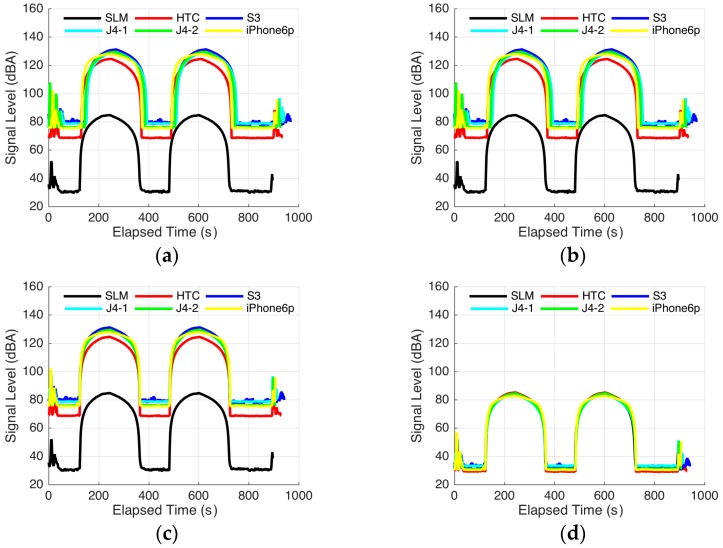
Calibration steps. (**a**) Raw time series from sound pressure level data from the SLM and phones; (**b**) Time series after resampling; (**c**) Time series after alignment; (**d**) Time series after calibration.

To facilitate the calibration process, we need a quiet room to perform our calibration. The other special equipment is CEM DT-8852, an SLM that conforms to the IEC 61672-1 Class 2 standard, which serves as a calibration standard. Several mobile phones are used to verify the calibration methods: an HTC butterfly J (HTC), a Samsung Galaxy I9300 S3 (S3), two Unistrong J4 (J4-1, J4-2), and an iPhone 6 plus (iPhone 6p). HTC and S3 are equipped with additional microphones. The calibration steps are described as follows:

*Step* *1:* Data recording. In a quiet room, while the calibration tone is being played, the sound pressure level [expressed in A-weighted decibel (dBA) unit] is recorded by the SLM and mobile phones. After this process has started, the operator leaves the room to prevent his actions or breathing from being recorded. Figure 2a shows that the segments with peaks are sinusoidal tones, and the duration between them represents silence. The data are recorded every 2 s.
*Step* *2:* Resampling. Because of the limitation of the SLM, we cannot substantially increase the sampling rate. As mentioned in the previous step, 2 s is selected. To improve the alignment precision in the follow-up step, as shown in Figure 2b, we employ 20 times the rate of the original signal for resampling by considering the balance between precision and computing load.
*Step* *3:* Time-series alignment. The calibration of the phones requires manual pressing of the buttons of the SLM or the mobile software to start the data recording. Simultaneously pressing the buttons of the different devices requires different processes, especially for products such as the CEM DT-8852 SLM. An unaligned time series will reduce calibration precision; thus, we need to align these data using algorithms. First, the correlation coefficients at each indentation between the resampled time series from the SLM and that from the phones are calculated. Then, we choose the indentation with the maximum correlation coefficient as a standard to translate the time series of the phones. In this manner, aligned time series are obtained, as shown in Figure 2c.
*Step* *4:* Calculating offsets. The duration of the calibration tone is 10 min. To obtain a complete time series, we need to start the recording before the tone is played and stop the recording for a period of time after completion. The pressing of the buttons of the SLM or mobile software by the operator and his other actions will be recorded during the time the tone is not played. To remove the noise from these actions, we introduce a sliding time window. The length of the window is the same as that of the tone (10 min). After calculating the means and standard deviations of the difference between the measurements of the sound pressure level by the SLM and the phones in the time window in each indentation, we choose the mean with the minimum standard deviation as offset *C* to compensate the measurements of each individual phone. Through this process, we can remove the noise caused by the operator and can calibrate the phones. The time-series compensated offset is shown in Figure 2d.

In these steps, the operator only plays the tone and records the data. The other processes are performed by the program. In this manner, we can significantly reduce the calibration workload. Another advantage is that the suitability of the phone to perform sound pressure level measurement can be evaluated by analysing the correlation between the time series in the SLM and the phones. Apparently, phones with low correlation coefficient are not suitable for sound pressure level measurement. The results of the experiment are presented in Section 4.

Figure 2 shows that large differences occur between the measurements from smartphones without calibration and the SLM under the same environment. These differences can be observed not only between the smartphones and SLM but also among smartphones of different brands and different phone ages. We observe that even for the same phone model, the calibration offset varies from phone to phone. Other research works have reached similar conclusions [13,27]. Therefore, for environmental noise measurements using smartphones, we need to individually calibrate each device.

### 2.3. Noise Mapping

After the phone calibration, we can use these phones to measure the sound level as well as the location and timestamp. Then, we can combine these data to generate the noise map. Because noise maps are constructed using GIS to integrate the measurements with the spatial information and infinitely increasing the spatial density of the measurements is impossible, we need a suitable interpolation method.

Tobler’s first law of geography states that all attribute values on a geographic surface are related to one another. Nevertheless, closer values are more strongly related than the distant ones [28]. However, spatial object types such as buildings, roads, and other objects are present in an area that requires a noise map. The spatial distribution of environmental noise is very complex, and spatial heterogeneity exists in environmental noise data [29].

**Figure 3:**
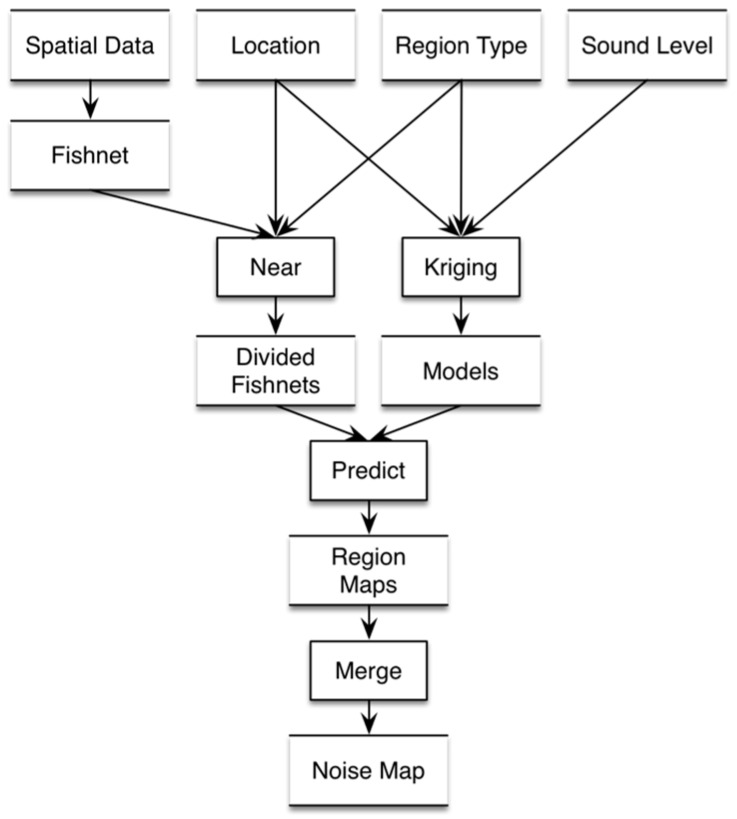
Steps in creating a noise map using the measurement and spatial data.

Several interpolation methods are available, such as the linear, nearest neighbour, and Kriging methods [30]; however, these interpolation methods do not take into account the influence of spatial heterogeneity on the interpolation results. A large difference in the value of the property between different sub-regions may exist; thus, the property values of two adjacent points are also different. Therefore, we design a noise-mapping method called region-based noise-mapping method (RNMM), which is based on the noise distribution of different regional types tagged by volunteers to model, interpolate, and combine these noise distributions to reduce the influence of spatial heterogeneity and improve the noise-mapping precision, as shown in Figure 3.

**Figure 4:**
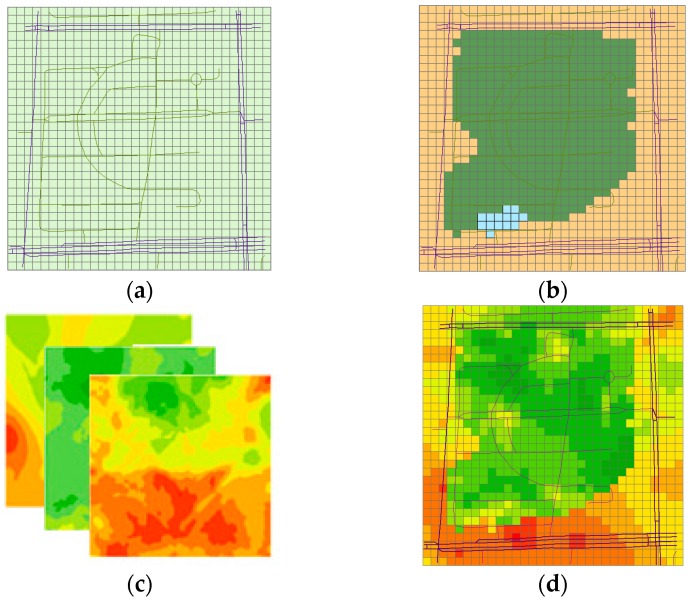
Diagrammatic sketch of steps of RNMM. (**a**) Create the fishnet with labels; (**b**) Nearest measurement and split into different region types; (**c**) Interpolation for different types; (**d**) Predict and combine the region types.

*Step* *1:* Create fishnet (grids) according to the extent of the experimental field. As is shown in Figure 4a.
*Step* *2:* Compute the nearest measurement for each grid and classify the grid into different types according to the label of the measurement (tagged by the volunteers). As is shown in Figure 4b.
*Step* *3:* Establish the spatial interpolation models of the noise for different region types. As is shown in Figure 4c.
*Step* *4:* Predict the sound level for the different region types based on the models established from the last step and create the noise maps for each type.
*Step* *5:* Combine the noise maps of the different region types to generate the noise map of the whole experimental field. As is shown in Figure 4d.

We follow the concept of participatory sensing based on the classification of the region tagged by the volunteers. The volunteers can easily mark the region types in the measurement; thus, these data can be easily obtained.

Ordinary Kriging was used for the spatial interpolation. The Kriging method is one of the most widely used geostatistical interpolation methods. Some related research activities also use this method in noise mapping [7,13]. Ordinary Kriging, one of the Kriging methods, is adopted in the present study for spatial interpolation of the noise data to measure the noise distributions. Kriging interpolation is applicable to regional variables with spatial correlation, and the spatial correlation is used for interpolation or extrapolation. It has a solid statistical theory basis and can estimate an error point by point, which is very suitable for the present study.

In summary, this method combines the subjective perception of the participants with the spatial interpolation method. It can fully utilise the advantages of the volunteer perception of the environment, and no additional new sources of data are required. Thus, it is a very practical and valuable method. In the next sections, we will verify this method by experiment.

## 3. Experiment

In this section, the devices used in the experiment are listed, and the experimental processes are outlined.

### 3.1. Devices and Software

**Figure 5:**
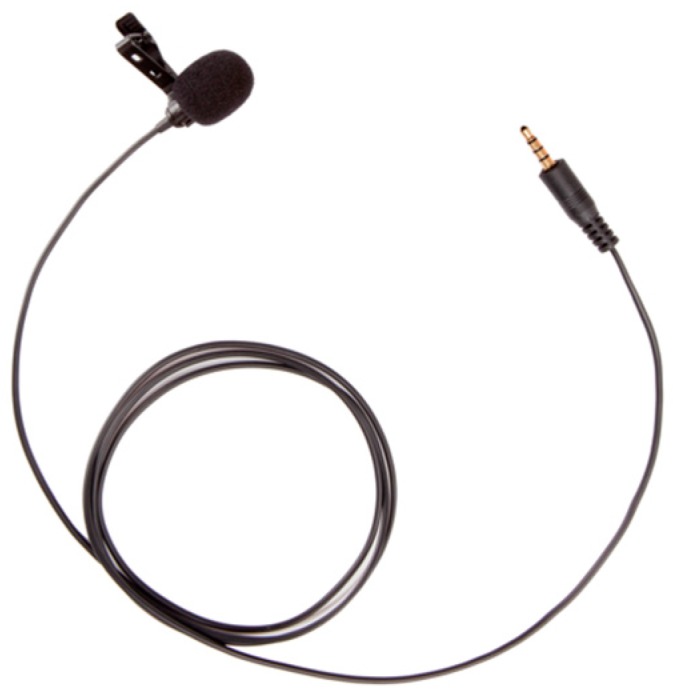
BY-LM10 microphone with a lapel clip and a foam windscreen.

As mentioned in Section 2.2.3, we use the IEC 61672-1 Class 2 SLM CEM DT-8852 (which costs about $170) as the calibration standard sound level in our experiment. Several mobile phones are used in the measurements: an HTC butterfly J, a Samsung Galaxy I9300 S3, two Unistrong J4, and an iPhone 6 plus. The additional microphone type is BY-LM10 (Figure 5), which is an omnidirectional condenser microphone specially designed for IPhone/IPad, IPod touch, and most Android devices [31]. Its price is no more than $20.00.

We wrote the measurement software used by the smartphones by ourselves. Matlab [32] and ArcGIS [33] were used to draw the figures.

### 3.2. Experimental Field

**Figure 6:**
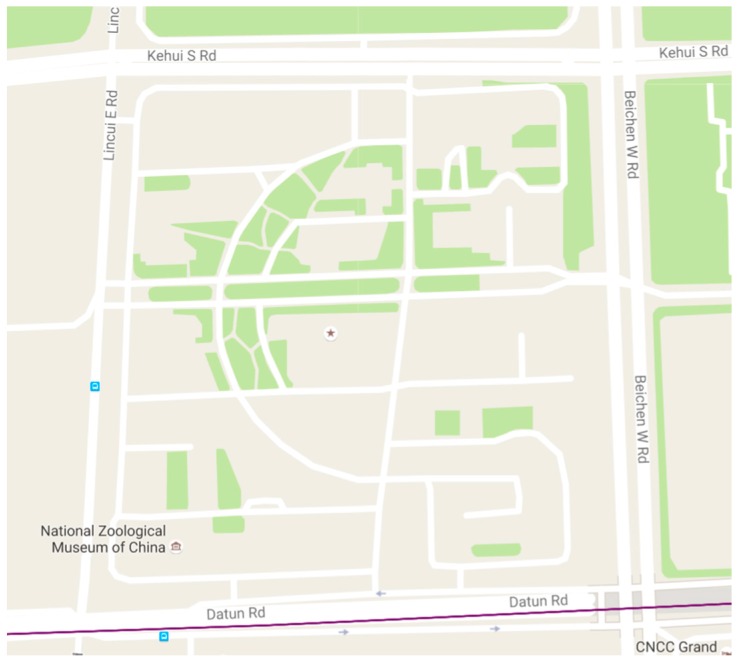
Experimental field: an office park of the Chinese Academy of Sciences in Beijing (from Google Map).

We choose an approximately 500 m × 500 m park in Beijing as our experimental field. The main plots and road distributions are shown in Figure 6 and described as follows:

This field is a typical area that includes a built-up region and a relatively empty region and is surrounded by roads in the four directions. As the main road, the southern part has a large traffic flow, and the other roads have relatively less traffic. We choose this area as our experimental field for two reasons. First, this field contains all the three typical regions we need (we need to use buildings, squares, and roads to represent this three typical regions). Second, the total size of the field meets our demand to generate sufficient measurements.

### 3.3. Experimental Process

In this study, the experimental process can be mainly divided into three steps:

*Step 1: Calibration*. The method mentioned in Section 2.2.3 is used to calibrate the phones and determine the precision. This part of the experiment is mainly completed in the room. In addition, the effectiveness and necessity of an additional microphone, together with the measurement results, are further studied.

*Step 2: Environment test*. The calibration work is done in the room. However, we need to determine whether the calibrated equipment reliably works in outdoor environment. In traditional noise monitoring, the noise meter should be placed in a fixed position. In contrast, in using smartphones for environmental monitoring, the volunteers neither stay in one place for a long time nor carry the equipment such as the tripod fixed to the mobiles. We need to determine whether the effect of wind resulting from the movements and other actions of the volunteers when they are walking fast or riding a bike for data collection can be neglected. We also need to determine whether the additional microphone with a wind shield can overcome the effect of wind on the measurement. These problems are addressed in the next sections.

To determine the effects of wind and motions such as walking and bicycle riding on the noise measurement, we use the SLM, HTC with a microphone and S3 without a microphone to measure the environmental noise under the conditions of walking and riding a bicycle. We perform a comparative analysis of the difference between these time-series measurements.

*Step 3: Measurement campaign*. In classic participatory sensing, the measurement process mainly relies on the spontaneous behaviour of the participants, and the measurement position is distributed with the behaviour of the participants [12,17]. This process inevitably results in an uneven distribution of measurement data in locations where the participants frequently pass by, thus generating more measurements. On the other hand, in locations where the participants seldom pass, few measurements are obtained, and some locations even have no data. In this study, we mainly rely on the planned campaign measurement to collect data: two volunteers (the volunteers are compensated and supported by our research fund) perform the measurement campaign twice during peak hours (8:00–9:00) and off-peak hours (21:00–22:00) on a work day to measure the sound levels covering the whole area of the office park and surrounding roads. To cover the whole area of the field, the volunteers have to walk around the field follow the same route in morning and night in order to ensure comparability of the results. When they walk along the road, they choose the road tag by using our software; when they go through the building area, they just choose the building tag, and when they go through the squares, they just choose the square tag. Volunteers can pin the microphone to the collar and can put the phone in the pocket. The software will tag automatically for each collect point until the volunteer change it, so the volunteer won’t be so tired. During the acquisition process, the record frequency of the A-weighted data is 2 s. Simultaneously, the three region types, namely, building, square and road, are tagged.

## 4. Results and Discussion

This section describes the measurement analysis and the noise-mapping results.

### 4.1. Noise Measurement

#### 4.1.1. Calibration and Measurement Precision

We employ the method mentioned in Section 2.2.3 to calibrate the smartphones. To verify the calibration results, we record and compare the corresponding results from the SLM and the phones under different sound levels. The results are listed in Table 1.

Table 1 indicates that in most cases, the measurements by the smartphones and SLM are very close. To more accurately describe the errors in measurements, Table 2 lists the measurement statistics.

From Table 2, we can make the following conclusions:

1. The differences in measurements between HTC, S3, J4-1, and SLM are relatively small; even the maximum difference is less than 1 dBA. We believe that these phones are suitable to carry out measurement of environmental noise.
2. The differences in measurements between J4-2, iPhone 6p, and SLM are slightly large but are much better than those of the uncalibrated devices.
3. Whereas J4-1 and J4-2 are of the same type and brand, differences exist in the calibration, which proves once again that independent calibration of each phone is necessary.
4. Although HTC and S3 are equipped with additional microphones, no significant difference is observed compared with the other phones.

#### 4.1.2. Additional Microphone

**Figure 7:**
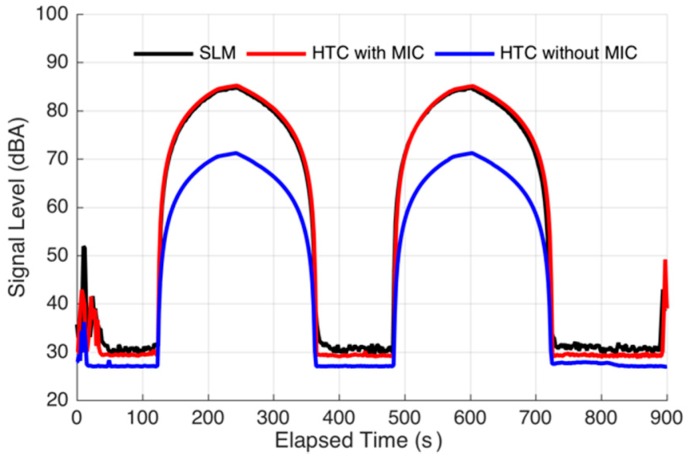
Comparison of the time series from HTC butterfly with and without an additional microphone.

To determine the validity of the calibration of a certain phone with or without an additional microphone, we use the offset from HTC with an additional microphone to calibrate the same phone without an additional microphone. The result is shown in Figure 7.

Figure 7 shows that the curve from the HTC with an additional microphone very well coincides with that from the SLM, which verifies the effectiveness of the calibration. However, although the shapes are similar, the curves from the HTC without any additional microphone appear very different from those of the SLM. In particular, when the sound level is higher than 60 dBA, the maximum difference is more than 10 dBA. This phenomenon can be quantised using the correlation coefficients. For example, Figure 7 shows that the correlation coefficient between the HTC with an additional microphone and SLM is 0.9792, and that between the HTC without an additional microphone and SLM is 0.8951, which is lower than all the other values shown in Figure 2d. Therefore, we do not consider that the HTC without any additional microphone is suitable for measuring environmental noise. Using the accumulated data, we can determine the threshold of whether the phones should be calibrated using the correlation coefficient between the phones and SLM. In this manner, we can also determine whether a phone is capable of measuring environmental noise without learning the details of the phone technical parameters.

#### 4.1.3. Effects of Wind and Movement

**Figure 8:**
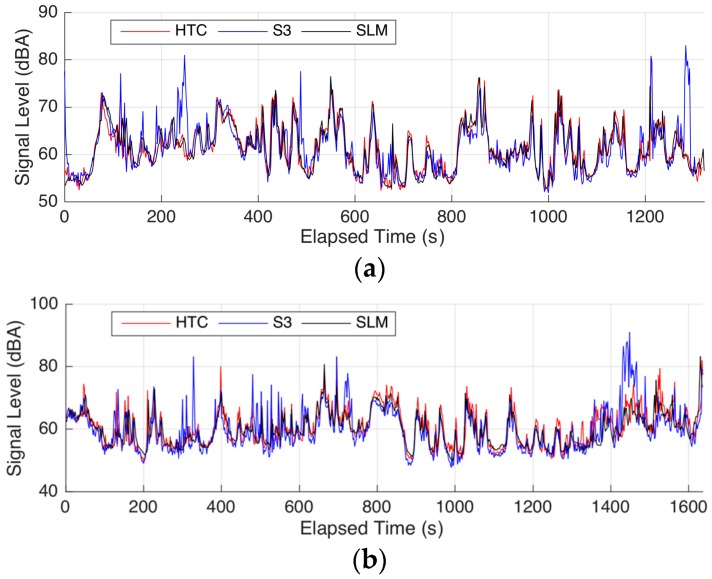
Time series of the measurements under different conditions: (**a**) walking and (**b**) cycling.

To ensure the necessity and effectiveness of additional microphones with a windscreen, we carried out a series of experiments. The DT-8852 SLM, HTC with an additional microphone, and S3 with built-in microphone were used in the experiments. The environment sound level was recorded using these three devices when a volunteer was walking or cycling. From the local weather forecast, the wind speed was less than 5 m/s. Figure 8 shows the time series of the data.

Figure 8a shows that the time series of the sound level from the HTC with an additional microphone (red line) is highly similar to that from the SLM when the volunteer was walking. The time series of the sound level from S3 with built-in microphone shows some unexpected fluctuations compared with that from the SLM. The correlation coefficient between the former two conditions was 0.8437, whereas that of the latter was 0.6894.

Similar phenomenon was observed when the volunteer was cycling, and the corresponding values were 0.7881 and 0.6756. We believe that the lack of windscreens for the built-in microphones is a major cause of these fluctuations. Therefore, we recommend that when environmental noise is measured, the result can be made more accurate using mobile phones equipped with an additional microphone with a windscreen. These additions not only reduce the effect of wind but also bring convenience to the users in that the microphones can be kept in the clothing, bag straps, and pockets. In this manner, we do not need to hold the phones in the hands for a long time, which eases the workload of the measurements. Of course, the mobile phones and microphones should be tested before the measurement using the methods presented in the previous section.

### 4.2. Noise Mapping

In the next sections, we present a rough description of the measurements and pay attention to the difference in the interpolation result using the RNMM and ordinary Kriging method.

#### 4.2.1. Noise Maps

The volunteers performed the measurement campaign twice during the peak hour (8:00–9:00) and off-peak hour (21:00–22:00) by covering the whole area of the office park and the surrounding roads. After eliminating the error data and exception handling, we obtained 1898 measurements during the peak hour and 2045 measurements during the off-peak hour. Then, we employed the method mentioned in Section 2.3 to deal with the data and created the noise maps. To obtain a balance between the computed load and map details, a 20 m × 20 m grid was chosen.

For RNMM, we take 10% as the validation data which is used for validation and 90% as the training data which is used to interpolation for each region type. For the Kriging method, we merge the three region types as the raw data, 10% of the raw measurement data as the validation data used for validation and 90% as the training data which is used to interpolation. 

**Figure 9:**
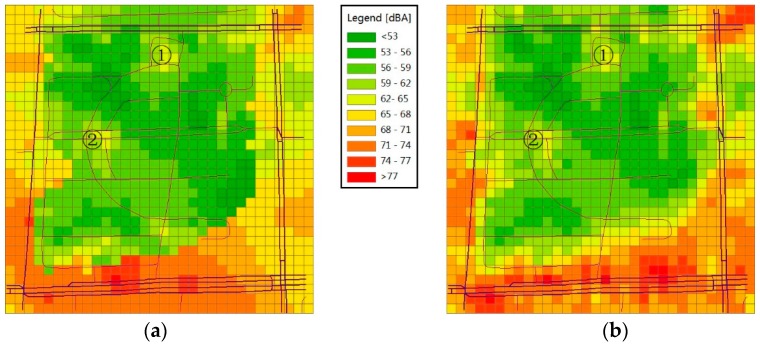
Noise maps of the experimental field during the peak hour (8:00–9:00) obtained by different methods: (**a**) RNMM and (**b**) Kriging.

**Figure 10:**
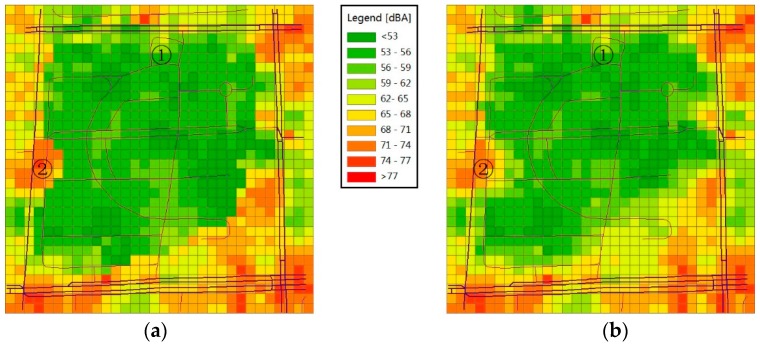
Noise maps of the experimental field during the off-peak hour (21:00–22:00) obtained by different methods: (**a**) RNMM and (**b**) Kriging.

The results for the peak and off-peak hours are shown in Figure 9 and Figure 10, respectively, where (a) uses the RNMM and (b) uses the Kriging method. By visual comparison of the two maps shown in Figure 9, we can see that the noise distribution of the two corresponds to the actual situation. The main road in the south shows higher sound levels, especially the crossroads in the southeast followed by the roads around the park. The sound levels in the area near the road tend to be higher than those far from the road. Most parts of the park display low sound levels except the area in the north (marked by ①) and that west of the central part (marked by ②). In the former, we believe that these higher values are partly due to the north exit of the park and the operation of large exhaust fans attached to a nearby dining hall. In the latter, we attribute the higher noise level to the intersection in the park where the crowd and vehicles generate loud noise.

In addition to the overall similarity, some marked differences are observed between the two maps. Figure 9b shows an obvious yellow area between the red area of the main road in the south and the green area in the park. We designate this yellow area as the transition zone. However, Figure 9a does not show such transition zone. In fact, many tall buildings are present in this area. The buildings will cause a dramatic change in the noise level. Therefore, we consider that the transition zone should not be obvious here compared with that shown in Figure 9b knowing that the Kriging method is based on spatial correlation. Hence, the interpolation produces a smooth transition without considering the spatial heterogeneity of sound propagation in the different region types. On the other hand, the different region types are modelled and interpolated by the RNMM by considering the influence of spatial heterogeneity.

The overall distributions of the sound levels of the two maps shown in Figure 10 are also similar. The road area still indicates a higher sound level; however, because the traffic is not very heavy at night, the noise level also decreases. Similar to the map during the peak hour, the crossroads at the four corners display higher sound levels. Without the influence of the pedestrian, only the noise of the exhaust fans operating for 24 h is present; thus, the yellow area in the north part of the park (marked by ①) also significantly decreases. The yellow area that indicates higher sound level inside the park no longer exists. A high-noise area indicated by red appears at the west side of the park (marked by ②) because some vendors gather here in the evening, causing a large concentration of people and thus increasing the noise level.

By comparing the two maps, we can still see a transition zone in Figure 10b. However, it occurs during the off-peak hour; thus, the difference in the noise levels between adjacent regions is not as obvious as that shown in Figure 9b. Therefore, transition zones only appear between adjacent regions where a large gap in the noise levels exists.

By analysing Figure 9 and Figure 10, we believe that measuring noise and creating noise maps using calibrated smartphones are feasible, and the noise-level distribution in the experimental field generated by this method is consistent with common sense. Some similarities are also observed between the RNMM and the Kriging method. With respect to the precision of the two methods, we present the issue in the next section.

#### 4.2.2. Precision Analysis

We have visually learned the difference in the interpolation results of the RNMM and Kriging method by comparing the noise maps presented in the last section. However, we have yet to determine the precision of the two methods. Because no official or other authorities in noise maps are available in the field, we cannot compare our map with an official one to determine its precision. Therefore, we use cross validation to determine the precision of spatial interpolation and compare their respective results. The results are listed in Table 3.

In Table 3, Kriging and RNMM respectively represent the statistics of the interpolation results of the whole area using the ordinary Kriging method and RNMM. Building, Road, and Square represent the statistics of the value of the three different region types using the ordinary Kriging method.

Table 3 indicates that the data during the peak or off-peak hour, the average error, and the standard deviation of the RNMM are smaller than those of the ordinary Kriging method, which prove the validity of the RNMM. We also note that the average error and standard deviation for the road, building, and square show a large difference. We argue that this difference is due to the spatial heterogeneity of the noise distribution in different region types.

Table 3 shows that the average error and standard deviation of the square area during the peak hour are larger than those during the off-peak hour mainly because during rush hour, a large number of people gathered in the square when the volunteers went through the crowd. Thus, noisier sounds were recorded which were largely random and uncertain, thus leading to a larger error and higher instability. Compared with the same place during the off-peak hour when the square was almost deserted, the results were very much different. Similarly, the building and road during the peak hour have a continuous balance of traffic flow and result in a relatively stable noise level. For the building region during the off-peak hour in the evening when fewer people are present and is relatively quiet, a sudden voice causes larger fluctuation in the collected data. For the road region, the distribution of traffic flow is not balanced, which increases the randomness of the noise distribution of the collected data points. Therefore, in these two regions, the standard deviation (or the volatility) during the off-peak hour was higher than that during the peak hour.

In summary, the RNMM has better spatial interpolation precision than the ordinary Kriging method by taking into account the difference in the spatial correlation in different region types. We believe that the RNMM is particularly suitable for participatory sensing data measurement using smartphones. Because the RNMM considers the participant understanding of the spatial distribution in the measurement region, the interpolation precision is further improved without increasing the workload or adding further information.

## 5. Conclusions

In this paper we have presented a noise-mapping method using smartphones, which involves two aspects: easily and effectively measure environmental noise and creating of a noise map of the whole area using limited measurement data.

To achieve certain precision for the measurement, we designed a set of methods to calibrate the smartphones. Using this method, we could easily complete the calibration without using additional equipment. We calibrated several common smartphones in our study. The result shows that most of the phones have measurement results close to that of the SLM after calibration. The error of the different phones largely varied even for the same types of phone, where the calibration value was still different. Therefore, we believe that individual calibration of each phone is necessary.

Using smartphones for noise monitoring is different from the traditional static observations. The phone users can be moving at any time, and holding phones with their hands to measure noise can bring great inconvenience to the users. At the same time, the influence of wind caused by the user movement and travel cannot be ignored. Therefore, we proposed a method and confirmed the necessity of attaching an additional microphone with a windscreen to reduce the effect of wind. The result shows that the additional microphone can effectively reduce the effect of motions such as walking and cycling on the measurement and can yield a measurement value closer to that of the SLM. We believe that in using smartphones for noise measurement, we need to attach an additional microphone, which can not only improve the precision of the measurement but also be more convenient to the users.

During the noise measurement, the density of the data that we gathered is always limited; thus, covering an entire area was impossible. Therefore, we need to achieve a full coverage of the measurement field using an interpolation method. The noise-level distribution can be locally abnormal as a result of spatial heterogeneity. To minimise the influence of spatial heterogeneity and to improve the precision of the local noise distribution in the noise map, we proposed the RNMM, which is based on the distribution of noise in different region types tagged by volunteers, to model, interpolate, and combine these distributions. We analysed the interpolation results of the data from the experimental field using the RNMM and ordinary Kriging. The result shows that the RNMM is more accurate in reflecting the local distribution of noise and consequently has better interpolation precision.

In this paper, We mainly focus on the participate sensing and citizen involving and try to measure sound levels and noise mapping by using the embedded sensors of mobile phones. In the following study, we will try to adopt the sound propagation models and simulate distributions using commercial software such as Cadna, SoundPLAN to improve our model and method continuously.

In summary, we believe that the proposed noise-mapping method based on smartphones is a fast and low-cost noise-mapping solution that can achieve certain precision and has practical value for cities that lack basic equipment for noise monitoring and for volunteers concerned about the urban environment. In the future, we intend to combine urban land-use maps and conduct further detailed study of the noise propagation characteristics in other region types such as an urban green space and to continuously enhance our proposed RNMM.

## Figures and Tables

**Table 1:** Measurements by SLM and smartphones at different sound levels (dBA).

SLM	HTC	S3	J4-1	J4-2	iPhone6p
53.75	54.31	54.60	54.34	56.38	56.10
63.20	63.57	63.89	63.68	65.82	65.54
67.56	67.87	68.22	68.02	70.12	69.87
70.24	70.61	70.98	70.80	72.95	72.64
72.41	72.59	73.00	72.83	74.99	74.67
74.00	74.15	74.58	74.43	76.58	75.89
76.99	77.01	77.51	77.36	79.50	77.61
80.65	80.57	81.05	80.88	83.01	79.69

HTC and S3 are equipped with additional microphones.

**Table 2:** Statistics of measurements by the SLM and smartphones (dBA).

	HTC	S3	J4-1	J4-2	iPhone6p
Mean	0.24	0.63	0.44	2.57	1.65
Maximum	0.56	0.85	0.59	2.71	2.40

**Table 3:** Mean and standard deviation of the different methods and different region types.

Period	Precision	Kriging	RNMM	Building	Road	Square
Peak hour	Mean	0.44	0.17	0.46	0.08	−2.19
	Std.	3.06	2.49	2.19	2.52	4.42
Off-peak hour	Mean	−0.09	−0.01	0.08	−0.02	−0.70
	Std.	3.11	3.02	2.50	3.46	1.07
